# Recovery of Complete Blindness and Internal Ophthalmoplegia After Transsphenoidal Decompression of Pituitary Apoplexy

**DOI:** 10.7759/cureus.28681

**Published:** 2022-09-01

**Authors:** Ravneet S Rai, Samuel Gelnick, Howard Pomeranz, Rashmi Verma

**Affiliations:** 1 Department of Ophthalmology, Northwell Health, Manhasset, USA

**Keywords:** amaurosis, ophthalmoplegia, blindness, apoplexy, pituitary

## Abstract

We report the case of a 64-year-old male who developed sudden onset of bilateral no light perception vision and bilateral total internal ophthalmoplegia after pituitary apoplexy. He underwent transsphenoidal pituitary decompression. Four months after the surgery, the patient recovered excellent functional vision (20/25) in one eye, though with significant residual visual field loss. He regained full extraocular motility bilaterally.

## Introduction

Pituitary apoplexy involves the sudden expansion of a pituitary mass, typically an adenoma, due to either hemorrhage or infarction. The most common visual disturbance is bitemporal hemianopsia due to mass effect on the optic chiasm, though a range of visual presentations can be seen [1]. Ophthalmoplegia can arise due to compression of cranial nerves III, IV, and VI. Clinical outcomes after pituitary apoplexy can vary significantly depending on the severity of the initial presentation and treatments performed [2,3]. While good outcomes have been demonstrated in cases of both early and late surgical intervention, the literature generally supports early intervention, particularly in cases of severe visual compromise [2].

## Case presentation

A 64-year-old male, not on any blood thinners, with a history of diabetes mellitus, hypertension, hyperlipidemia, polysubstance abuse, and no ophthalmic history, presented to the emergency department with a four-day history of worsening headache and one-day history of nausea, vomiting, and blurry vision in both eyes. There was no history of eye pain, head or ocular trauma, recent infection, or sick contacts. The patient was lethargic, uncooperative, and was not alert or oriented to time, place, or person. The initial ophthalmic examination was notable for bilateral blink-to-light vision, inability to maintain gaze or track, sluggishly reactive pupils, and bilateral complete restriction of eye movements in all gazes. Intraocular pressures were 15 mmHg OD and 17 mmHg OS. The anterior segment examination was unremarkable. Dilated fundus examination showed cup-to-disc ratios of 0.65 and 0.70 of the right and left optic nerves, respectively, and temporal pallor of both optic nerves.

**Figure 1:**
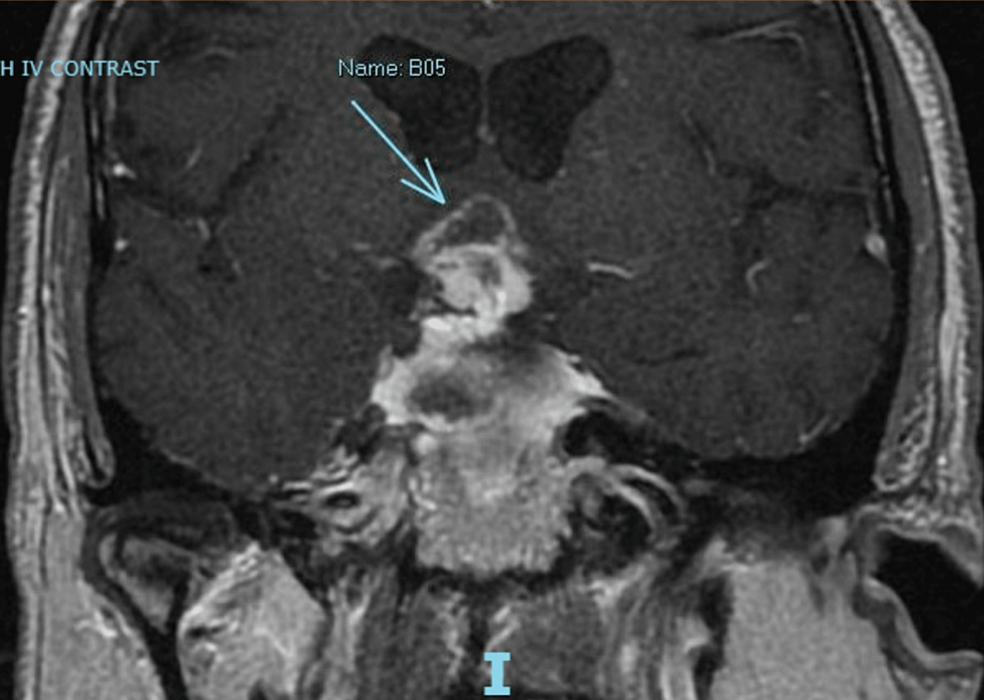
Sellar mass with suprasellar extension (blue arrow), with intrinsic hemorrhagic components and peripheral enhancement.

Computerized tomography (CT) scan revealed a sellar mass with suprasellar extension. Due to the patient’s agitation and combativeness, he was unable to tolerate magnetic resonance imaging (MRI). Laboratory testing revealed normal follicle-stimulating hormone, low luteinizing hormone, low prolactin, normal thyroid-stimulating hormone, normal free T4, low adrenocorticotropic hormone (ACTH), and low AM cortisol. ACTH stimulation testing was consistent with adrenal insufficiency likely secondary to the compressive effect from the mass. The patient was started on hydrocortisone 50 mg q8H. A lumbar puncture was performed to rule out meningitis given his severely altered mental status, and cerebrospinal fluid (CSF) analysis was negative for any infectious process. By day seven of admission, the patient’s vision had deteriorated to no light perception (NLP) OU. His pupils became amaurotic and he continued to have total internal ophthalmoplegia. Consent from his family for MRI under general anesthesia was obtained, and MRI under anesthesia was performed on day seven. MRI of the brain with and without intravenous (IV) contrast showed hemorrhagic components of the sellar mass with suprasellar extension and peripheral enhancement, highly suspicious for hemorrhagic pituitary adenoma. There was a mass effect on the anterior medial aspect of thalamic massa intermedia and vasogenic edema in bilateral aspects of the anterior commissural region. The mass extended into the cavernous sinuses, the left greater than the right (Figure 1).

On day nine of the admission, the patient underwent transsphenoidal pituitary decompression. Surgery was delayed until the ninth day of the admission due to the inability of the patient to tolerate MRI without anesthesia, as well as other investigations that were concurrently undertaken to explain the patient’s change in mental status. A biopsy of the mass taken during the surgery revealed pituitary adenoma with extensive necrosis, organizing hemorrhage, and granulation tissue, confirming the diagnosis of pituitary apoplexy. Approximately two weeks after admission, he had a slight improvement in downgaze OU, full recovery of adduction OD, and nearly full recovery of abduction OS. His hospital course was complicated by postoperative fever, for which he was treated with prophylactic antibiotics.

On outpatient examination four months after the surgery, his right eye remained NLP while his left eye had improved to 20/25. His extraocular movements had improved to full in both eyes. His right pupil remained amaurotic but his left pupil was briskly reactive to light. Humphrey visual field testing of the left eye revealed almost total loss of visual field except for a small central island.

## Discussion

Among patients with pituitary tumors of any kind complaining of vision loss, a measurable decrease in visual acuity has been reported in between 42% and 88% of patients [4]. Pituitary apoplexy involves the sudden expansion of a pituitary adenoma and can be either hemorrhagic or non-hemorrhagic. More than 50% of patients with pituitary apoplexy present with visual disturbance to some degree, though NLP vision is rare [1]. Headache and limitation of extraocular movements are seen in upwards of 80% and 50% of patients, respectively [1].

The incidence of binocular blindness in pituitary apoplexy is currently not well studied. In a series of over 2,000 patients with pituitary adenoma in China, 97 patients developed apoplexy, and five of them subsequently developed blindness, though the presence of monocular or binocular blindness was not specified [5]. In a series of 23 patients with pituitary apoplexy in India, two patients developed monocular blindness and six developed binocular blindness. Blindness was defined in this study as NLP. After transsphenoidal decompressive surgery, half of the blind eyes recovered some vision, i.e., four eyes to 20/600 or better and three to 20/20, at the final follow-up, which was at minimum three months [6].

Although transsphenoidal surgery can improve visual acuity in cases of pituitary apoplexy involving vision loss, the final outcome may be poorer in patients with blindness [7]. Patients treated conservatively generally have the same degree of improvement in visual function as those treated with surgery [8], although those treated conservatively may have less severe visual dysfunction to begin with [1]. The median time to visual acuity improvement is also similar between those treated conservatively and those treated surgically [8].

With regard to the timing of surgery, there is no significant difference in visual outcomes between early and late surgery. A recent meta-analysis analyzing 200 patients found that there was no difference in final visual outcomes in patients undergoing surgery within seven days or after seven days following apoplexy [9]. Similarly, in surgically treated patients, ophthalmoplegia may improve in patients with apoplexy even if surgery is delayed [10]. However, surgical outcomes for ophthalmoplegia (31-57% of patients experiencing recovery) are poorer than for loss of visual acuity (>80% of patients experiencing some recovery) [9]. Hence, our patient fell in the relatively smaller group of patients who experience some recovery of both visual acuity and ophthalmoplegia after surgery.

## Conclusions

We report a case of bilateral blindness and bilateral total internal ophthalmoplegia presenting after pituitary apoplexy, with partial recovery of vision and full recovery of motility following transsphenoidal surgery nine days after the apoplectic event. Our patient regained excellent central vision (20/25) in his left eye, though his visual field in that eye was severely restricted. He regained full ocular motor function. This case is an addition to the literature showing return of functional vision in a patient with a severe presentation of pituitary apoplexy who underwent surgery relatively late after the apoplectic event.
